# Clionasterol-Rich Fraction of *Caulerpa racemosa* against Particulate Matter-Induced Skin Damage via Inhibition of Oxidative Stress and Apoptosis-Related Signaling Pathway

**DOI:** 10.3390/antiox11101941

**Published:** 2022-09-28

**Authors:** N. M. Liyanage, D. P. Nagahawatta, Thilina U. Jayawardena, H. H. A. C. K. Jayawardhana, Hyo-Geun Lee, Young-Sang Kim, You-Jin Jeon

**Affiliations:** 1 Department of Marine Life Sciences, Jeju National University, Jeju 63243, Korea; 2 Department of Chemistry, Biochemistry, and Physics, Université du Québec à Trois-Rivières, Trois-Rivières, QC G8Z 4M3, Canada

**Keywords:** *Caulerpa racemose*, particulate matter, mitochondria, apoptosis, oxidative stress, clionasterol, zebrafish model

## Abstract

The increasing airborne particulate matter (PM) consisting of environmental contaminants such as dust, aerosols, and fibers has become a global concern by causing oxidative stress that leads to apoptosis and skin damage. The current study evaluated the protective effect of *Caulerpa racemosa* (CR) against PM-induced skin damage using human keratinocytes and a zebrafish model. The clionasterol-rich hexane fraction (CRHF2) of CR exhibited superior protective activity through downregulating intracellular reactive oxygen species levels and mitochondrial ROS levels, as well as the PM-induced increase in apoptotic body formation and upregulation of apoptotic signaling pathway proteins, along with sub-G1 cell accumulation dose-dependently. Furthermore, in vivo results showed that CRHF2 potentially downregulates PM-induced cell death, ROS, and NO production in the zebrafish model. Hence, the results evidenced that the protective effect of CRHF2 is caused by inhibiting oxidative stress and mitochondrial-mediated apoptosis in cells. Therefore, *C. racemosa* has the potential to be used in the development of pharmaceuticals to attenuate PM-induced skin diseases.

## 1. Introduction

Air pollution by environmental pollutants such as particulate matter (PM) is a matter of interest worldwide, particularly in China, Korea, and Japan. It has caused several deleterious effects on human health as well as regional and global climatic changes. Any toxic gas or airborne particles with a diameter less than 10 μm are included in air pollutants. They can be composed of organic or inorganic compounds or of both. The size, composition, and the origin of these particles are based on their microenvironment [1]. PM contains organic compounds that can readily penetrate the skin [2]. The skin is the largest organ in the human body and provides the largest interface between the body and external environment. Therefore, the emergence of skin diseases caused by external factors is common. Recently, the deleterious effects of PM on skin have attracted the attention of dermatologists and scientists worldwide [3,4]. PM results in oxidative stress injuries such as DNA damage and repair, cell apoptosis, and inflammation in skin keratinocytes [5,6,7].

Natural antioxidants present in the cells protect against injuries caused by reactive oxygen species (ROS). Superoxide dismutase, catalase, and glutathione transferase are primary enzymatic antioxidants, whereas low-molecular-weight ascorbic acids, α-tocopherol (vitamin E), β-carotene, and glutathione are important non-enzymatic antioxidants [8]. In addition, supplementation with commercially synthesized antioxidants such as butylated hydroxyanisole, butylated hydroxytoluene, and propyl gallate is being applied to prevent the adverse effects caused by excess ROS accumulation [9]. However, considering their synthetic nature, PM has been reported to have toxic and carcinogenic effects on humans. This has led to an increased interest in developing natural treatments that would be safe for humans.

Marine seaweeds are a valuable resource for important bioactive compounds, particularly phytosterols. These are lipid-rich compounds produced by plants and are the main lipid constituents of the biological plant cell membranes. Phytosterols isolated from algae possess antioxidant activity [10]. Clionasterol is a member of the class of phytosterols having a role as a marine metabolite. It is proven to possess antibacterial and antifungal activities [11]. It has also been tested for its influence on the classical and alternative pathways of the activation of the human complement system [12]. The activity of marine phytosterols in preventing skin damage was not studied extensively in the past.

Recently, marine seaweeds have gained attention as natural antioxidants and skin protectants for the production of pharmaceuticals and cosmeceuticals, owing to their availability and safety in long-term use. *Caulerpa racemosa* is a green marine alga distributed in warm waters in tropical regions. It is popular as a raw material for salads in most South Asian countries, including Sri Lanka. It is also used as an ingredient in folk medicine to treat hypertension and rheumatism. Due to their harsh environmental growth conditions, these seaweeds are exposed to extreme stress, leading to the production of free radicals and other oxidants [13]. In a previous study, it was shown that *C. racemosa* contains higher antioxidant and phenolic components than those in red seaweeds [14]. Although extensive studies have been conducted on various seaweed extracts, to the best of our knowledge, limited data are available on the antioxidant and antiapoptotic potential of *C. racemosa* and its application in pharmaceutical and cosmeceutical production. Hence, the present study aimed to explore the application of *C. racemosa* as a potential skin protective agent.

## 2. Materials and Methods

### 2.1. Materials

Human keratinocytes (HaCaT) were purchased from the Korean Cell Line Bank (KCLB, Seoul, Korea). Cell line was maintained in Dulbecco’s modified essential medium (DMEM, Gibco) with L-glutamine, 1% (*v*/*v*) antibiotics (penicillin, streptomycin), and 5% (*v*/*v*) fetal bovine serum (FBS) purchased from Gibco-BRL (Grand Island, NY, USA). The certified reference material (CRM) for PM (CRM No.28 Urban Aerosols) was purchased from the Center for Environmental Measurements and Analysis, National Institute for Environmental Studies (NIES), Ibaraki, Japan. Chemicals such as 3-(4,5-dimethylthiazol-2-yl)-2,5-diphenyltetrazolium bromide (MTT), 2′,7′-dichlorodihydrofluorescein diacetate (DCFH2-DA), acridine orange, Hoechst 33342, and Ethidium bromide were purchased from Sigma-Aldrich (St. Louis, MO, USA). Antibodies used in this study were purchased from Santa Cruz Biotechnology (Santa Cruz, CA, USA). Analytical-grade solvents such as hexane, ethanol, and methanol were obtained from Sigma-Aldrich (St. Louis, MO, USA).

### 2.2. Collection of Seaweeds and Extraction

**Figure 1:**
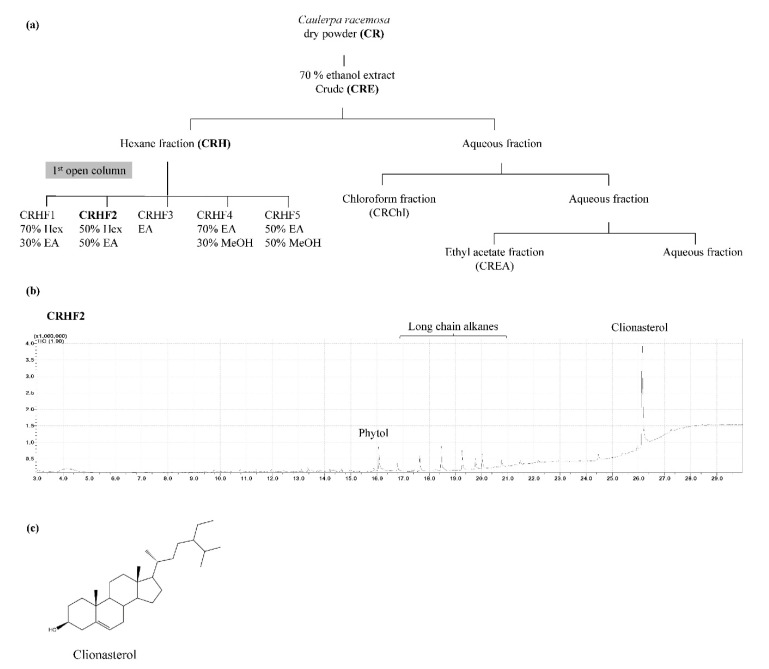
(**a**) Separation procedure implementing solvent/solvent extraction method leading to active fraction CRHF2, (**b**) GCMS chromatogram of CRHF2, (**c**) chemical structure of clionasterol.

The green seaweed *C. racemosa* was collected from coastal areas of Maldives and Sri Lanka. Samples were immediately washed to remove impurities. The washed seaweeds were dried using a hybrid hot water Goodle dryer [15] and lyophilized. Extraction was performed as previously described [16]. Briefly, 50 g of the ground sample was extracted with 70% ethanol to acquire *C. racemosa* ethanolic extract (CRE). It was suspended in deionized water and fractionated using hexane, chloroform, and ethyl acetate. The hexane fraction was further resolved into five fractions, CRHF1, CRHF2, CRHF3, CRHF4, and CRHF5, using a silica open column due to its potential bioactivities. Gas chromatography–mass spectrometry (GC/MS) analysis was performed to characterize the five fractions, from which CRHF2 was selected for further experiments due to the presence of a key compound, clionasterol. The bioassay-guided purification process is illustrated in Figure 1a. Fractions were evaporated using a rotary evaporator followed by freeze-drying.

### 2.3. CRM No: 28 for Particulate Matter

CRM was developed by the NIES to identify the elements in PM. Detailed characterization of PM was conducted in previous studies [17,18]. Their analyses were based on detailed spectroscopic analyses of atomic absorption spectroscopy, instrumental neutron activation analysis, particle-induced X-ray emission, and X-ray fluorescence. The size distribution analysis revealed that majority of the PM had a diameter of approximately 2 µm. Furthermore, it consisted of eight polycyclic aromatic hydrocarbons, and among them, the highest mass fraction was benzo[b]fluoranthene. The earth metals detected were magnesium, calcium, strontium, barium, and inorganic materials, while the transition metals detected were manganese and lead, which contributed to the higher mass fraction.

### 2.4. Cell Viability

HaCaT cells were cultured in DMEM supplemented with 10% FBS and 1% penicillin/streptomycin mixture at 37 °C, under 5% CO_2_ humidified atmosphere. PM stock solution was prepared by suspending the PM in DMEM and diluting when necessary. Cells were seeded (1 × 10^5^ cells/mL) in 96-well plates and incubated for 24 h. Column fractions (CRHF1-CRHF5) were added to cells at final concentrations of 25, 50, 100, and 200 μg/mL. After 2 h of incubation, cells were stimulated with 200 μg/mL PM. The working concentration of the PM to be treated was established based on preliminary studies. After 24 h of incubation with PM, cell viability was measured using MTT assay [19].

### 2.5. Intracellular ROS Scavenging Activity of PM-Induced Cells

DCFH2-DA assay was used to measure the intracellular ROS scavenging potential of the samples. A 96-well plate was seeded with HaCaT cells (1 × 10^5^ cells/mL) and incubated for 24 h. Cells were pretreated with CRHF2 for 2 h before stimulating with PM. The wells were treated with DCFH2-DA (25 µg/mL) for 10 min and fluorescence measurements (excitation—485 nm, emission—530 nm) were performed using a microplate reader (Bio-Tech, Winooski, VT, USA).

The superior antioxidant and protective activities of the CRHF2 fraction were further confirmed by flow cytometry using DCFH2-DA staining of cells [20]. After CRHF2 pretreatment and PM stimulation, the cells were washed and treated with DCFH2-DA for 30 min at 37 °C in dark. Flow cytometry (CytoFLEX, Beckman Coulter, PA, USA) was used to analyze the stained cells.

### 2.6. PM-Induced Cell Apoptosis

To determine cell apoptosis due to PM, the nuclear morphology of cells was evaluated using DNA dye Hoechst 33342 and nuclear double-staining method. HaCaT cells were seeded in 24-well plates and pretreated with CRHF2 (25 and 50 μg/mL) prior to PM stimulation for 24 h. Cells were treated with Hoechst 33342 at a final concentration of 10 μg/mL and further incubated for 10 min. Stained cells were observed under a fluorescence microscope (Olympus, Tokyo, Japan) and apoptosis levels were measured using ImageJ software [21]. A mixture of acridine orange and ethidium bromide was used for double staining of PM-induced HaCaT cells. After incubation for 10 min, cellular morphology was assessed using a fluorescence microscope equipped with CoolSNAP-Pro color digital camera [22].

### 2.7. Analysis of Cell Cycle

To identify apoptotic sub-G1 cells, flow cytometry analysis was performed according to a previously reported method [23]. Briefly, cells were seeded in 6-well plates, treated with CRHF2, and incubated for 12 h. Following this, the cells were harvested and fixed with 1 mL of 70% ethanol at 4 °C overnight. Fixed cells were then washed twice with cold phosphate saline buffer (PBS) and the supernatant was separated. The cells were further incubated in PBS containing propidium iodide, 100 μg RNase A, and ethylenediaminetetraacetic acid EDTA at 37 °C for 30 min. Cells were analyzed using a fluorescence-activated cell sorting (FACS) Calibur flow cytometer (Becton Dickinson, San Jose, CA, USA).

### 2.8. Mitochondrial ROS Generation Measurement

Mitochondrial membrane permeability is related to apoptosis through caspase-associated protein activation, and membrane permeability depends on mitochondrial oxidative stress [24]. Mitochondrial ROS generation in PM-induced HaCaT cells was analyzed using dihydrorhodamine-123 (DHR123) staining. Seeded cells were pretreated with CRHF2 and stimulated with PM for 24 h. the stimulated cells were incubated with 25 µM DHR 123 (Sigma-Aldrich, Poole, UK) for 20 min in dark conditions. Excess DHR123 was removed and cells were observed under a fluorescence microscope.

### 2.9. Western Blot Analysis

Western blotting was performed for the evaluation of intracellular expression levels of selected key molecular mediators related to apoptosis, including Bax, Bcl-xL, Caspase-3, P53, cleaved PARP, and cleaved caspase-9. Cells were seeded and treated with CRHF2 sample for 24 h, following incubation with PM. Finally, cells were harvested for protein extraction and proteins were analyzed using a BCA protein assay kit. Electrophoresis was carried out using 12% sodium dodecyl sulphate-polyacrylamide gels. Resolved bands were blotted onto nitrocellulose membranes and blocked with 5% skimmed milk in tris-buffered saline with 0.1% Tween 20. Membranes were consecutively incubated with selective primary and secondary antibodies and signals were developed using chemiluminescent substrate (Cyanagen Srl, Bologna, Italy). Fluorescence images were obtained using the FUSION SOLO Vilber Lourment system. ImageJ program was used for densitometric analysis of proteins.

### 2.10. In Vivo Antioxidant Activity of CRHF2 Using Zebrafish Model

Animal experiments were conducted in accordance with the experimental animal guidelines provided by Jeju National University Animal center and were authorized by the Animal Care Use Committee (IACUC) of Jeju National University (protocol 2020-0049).

#### 2.10.1. Maintenance of Zebrafish

Adult zebrafish were purchased from the Seoul Aquarium, Korea and maintained in acrylic tanks under controlled conditions (28 °C, 14/10 h light/dark cycle). Embryos were obtained by natural spawning and were collected within 30 min.

#### 2.10.2. Application of CRHF2 to Zebrafish Embryos

Zebrafish embryos were transferred to a 12-well plate (15 embryos/well) containing embryo medium within 7–9 h postfertilization. They were treated with CRHF2 (25, 50, and 100 μg/mL) and incubated for 1 h. Stimulation with PM (400 μg/mL) was performed and incubated for 24 h. The survival rate of PM-stimulated zebrafish was measured for 7 days. The heartbeat rate of stimulated zebrafish was recorded 2 days postfertilization (dpf) [25,26].

#### 2.10.3. Cell Death, Intracellular Lipid Peroxidation, and ROS Analysis

Cell death was evaluated using acridine orange staining (7 μg/mL) and intracellular ROS levels were measured using DCFH2-DA (20 μg/mL) at 3 dpf. The lipid peroxidation levels of PM-stimulated zebrafish were measured by diphenyl-1-pyrenylphosphine (DPPP) staining (25 μg/mL) [25].

### 2.11. Statistical Analyses

Quantifiable data are expressed as the means± standard deviation (SD), based on at least three independent evaluations (n = 3). One-way analysis of variance was used to compare mean values. *p*-values < 0.05 (*p* < 0.05), * and 0.01 (*p* < 0.01), ** were considered statistically significant.

## 3. Results

### 3.1. Characterization of CRHF2 Fraction of C. racemosa and PM

Based on the bioassay-guided evaluations of the five hexane fractions, CRHF2 was selected for further studies because of its superior activity. GC/MS analysis of CRHF2 identified two sterol compounds, clionasterol and phytol. Between the two, clionasterol in CRHF2 was identified as a key compound (Figure 1b). The chemical structure of clionasterol is shown in Figure 1c.

### 3.2. Protective Effect of Five Hexane Fractions of C. racemosa against PM-Induced ROS Generation

**Figure 2:**
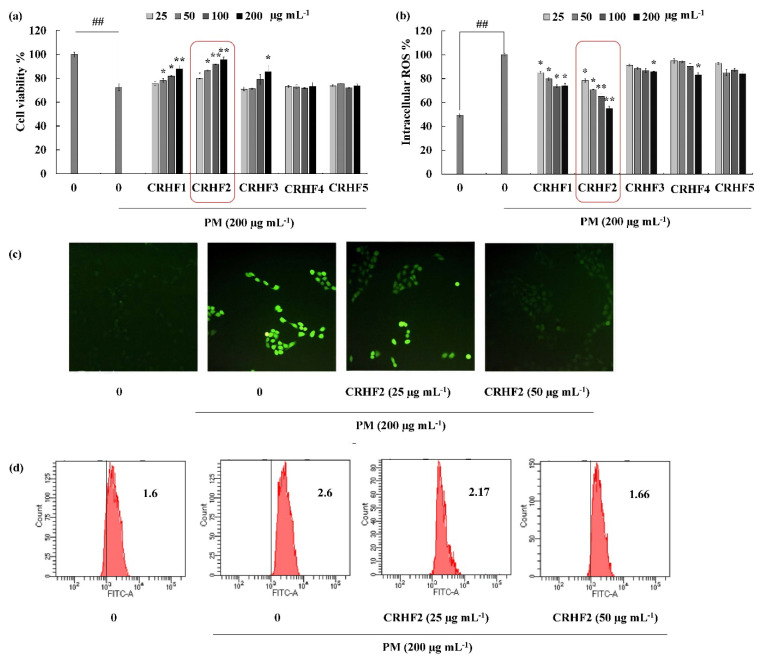
CRHF2 protects PM-induced oxidative stress in HaCaT keratinocytes. Effect of column fractions on (**a**) cell viability and (**b**) intracellular ROS levels, (**c**) fluorescence microscopy images, and (**d**) flow cytometry results of DCFH2-DA stained cells. The values in the histogram represent the mean intracellular ROS levels. Results are represented as mean ± SD of triplicate determinants (n = 3); * *p* < 0.05, ** *p* < 0.01. (# denotes significance compared to control while * represents significance compared to PM-treated group).

The cell viability and intracellular ROS in PM-induced cells were evaluated to investigate the antioxidant potential of hexane fractions isolated from the seaweed. As shown in Figure 2a, the cell viability of PM-stimulated HaCaT cells was drastically decreased compared to that in nontreated cells; however, treatment with the hexane fractions (25, 50, 100, and 200 μg/mL) increased cell viability in a dose-dependent manner. Similarly, PM significantly stimulated intracellular ROS generation in cells, whereas treatment with *C. racemosa* hexane fractions remarkably and dose-dependently downregulated ROS production and attenuated the effects of PM on HaCaT cells in treated cells (Figure 2b). However, among the five fractions, the CRHF2 fraction showed excellent ROS scavenging activity. Therefore, CRHF2 was used in further experiments.

Images for DCFH2-DA staining of cells showed antioxidative activity of CRHF2 in PM-stimulated HaCaT cells. According to the fluorescence microscopy images, an amplified intensity of green fluorescence was observed in PM-stimulated cells compared to that in the untreated group (Figure 2c). Upon treatment with CRHF2, the fluorescence intensity dose-dependently decreased. The superior antioxidant activity of CRHF2 was confirmed using FACS and DCFH2-DA fluoroprobe. Flow cytometry analysis of DCFH2-DA-stained cells indicated a peak shift towards higher intensity (X axis-FITC-A) due to PM stimulation of cells compared to that in the nontreated group (Figure 2d). However, this effect was reversed with increasing concentrations of CRHF2.

### 3.3. CRHF2 Attenuated PM-Induced Apoptotic Body Formation

**Figure 3:**
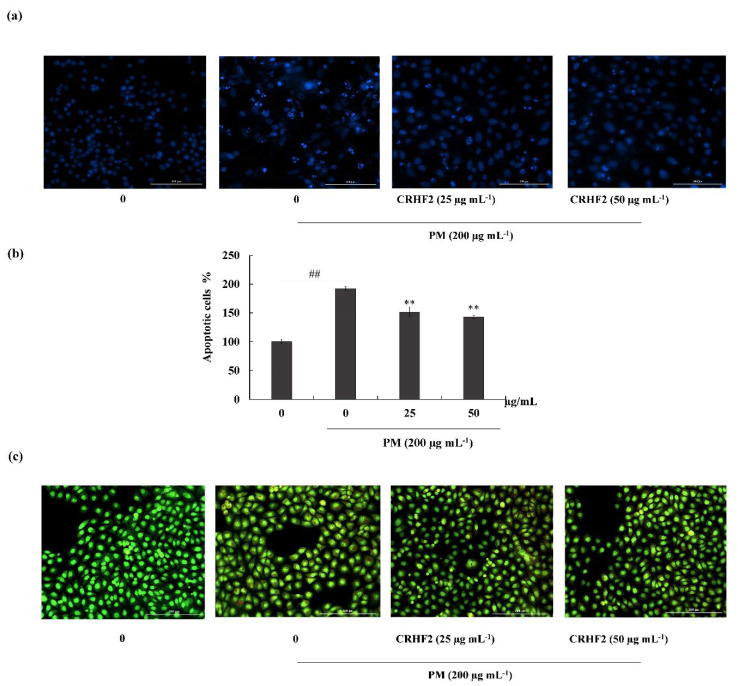
CRHF2 attenuates PM-stimulated apoptotic body formation and DNA damage. Evaluation of nuclear morphology via (**a**) Hoechst 33342 staining fluorescence images (**b**) apoptotic cell percentage, and (**c**) ethidium bromide and acridine orange-involved double staining. Experiments were triplicated to confirm their repeatability (n = 3), ** *p* < 0.01 (# denotes significance compared to control, while * denotes significance compared to PM-treated groups).

Hoechst 33342 staining and nuclear double staining with ethidium bromide and acridine orange were used in studying the nuclear morphology of the cells. Hoechst staining revealed an increase in chromatin condensation and DNA fragmentation in PM-stimulated cells compared to those in nontreated cells, representing a higher intensity in the nuclei region (Figure 3a). After dose-dependent treatment with CRHF2, the number of apoptotic bodies was significantly decreased, resulting in the decreased fluorescence intensity. This was proven with the percentage of apoptotic cell measurement with ImageJ software. As shown in Figure 3b, PM stimulation of HaCaT cells increased in the late apoptotic stages, as indicated with by orange-colored nuclei fragments upon double nuclear staining using ethidium bromide and acridine orange double staining (Figure 3b). Treatment with CRHF2 suppressed the appearance of apoptotic bodies in a dose-dependent manner, demonstrating its protective effect.

### 3.4. Effects of CRHF2 on Sub-G1 DNA Content and Late Apoptotic Event

**Figure 4:**
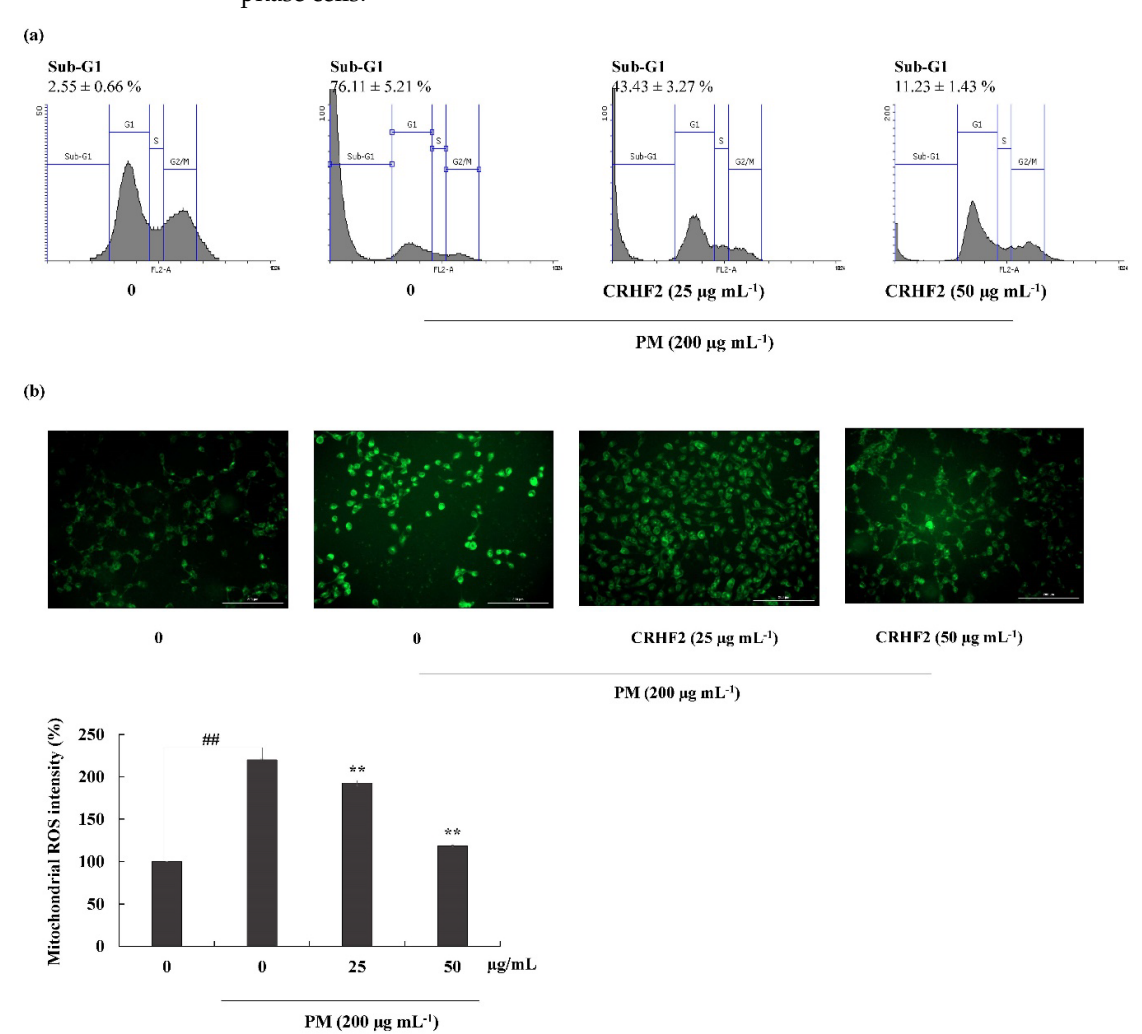
Cell-cycle analysis and mitochondrial ROS analysis in CRHF2-treated cells. (**a**) Cell-cycle analysis of sub-G1 cell population. PM affected cell-cycle progression and it was recovered with CHRF2 treatment. Cells were treated with CRHF2 and induced with PM. Harvested cells were evaluated via flow cytometer. (**b**) Mitochondrial ROS analysis by DHR123 staining. Triplicated independent experiments (n = 3) were involved to confirm the repeatability. Results are represented as mean ± SD; ** *p* < 0.01. (# denotes significance compared to control while * represents significance compared to PM-treated group).

Flow cytometric analysis was performed to quantify apoptosis. As shown in Figure 4a, the sub-G1 DNA content in the nontreated control and the PM-stimulated group were 2.55 ± 0.66% and 76.11 ± 5.21%, respectively, indicating an increase in the percentage of cells in the late apoptotic stage. However, this effect was improved by dose-dependent treatment with CRHF2, along with a substantial reduction in the proportion of sub-G1 phase cells.

### 3.5. Mitochondrial ROS Production was Ameliorated by the Treatment of PM-Induced Cells with CRHF2

DHR123 is a cell-permeable fluorogenic indicator of ROS levels, particularly in the mitochondria. The ROS generated in mitochondria oxidized DHR123 into rhodamine 123 and emitted green-color fluorescence (Figure 4b). Mitochondrial ROS is a major determinant of cell apoptosis. Based on the results obtained, PM stimulation resulted in higher fluorescent intensity, indicating overexpression of mitochondrial ROS; however, dose-dependent treatment with CRHF2 decreased fluorescent intensities, thus inhibiting the ROS generation.

### 3.6. Effect of CRHF2 on Mitochondria-Mediated Apoptosis Signaling in HaCaT Cells

**Figure 5:**
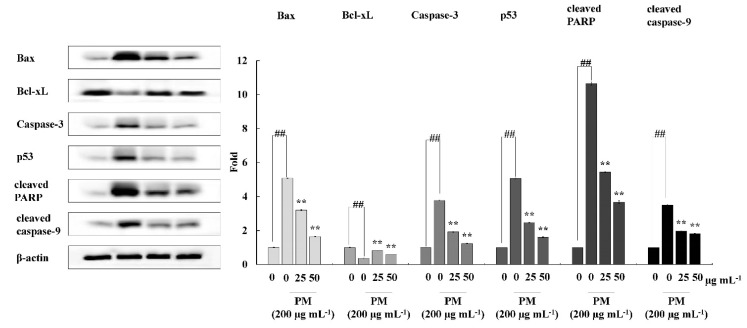
CRHF2 protects HaCaT keratinocytes from PM-stimulated apoptosis by inhibiting apoptotic proteins. Experiments were triplicated (n = 3). Results are represented as mean ± SD; ** *p* < 0.01. (# denotes significance compared to control while * represents significance compared to PM-treated group).

To investigate the mechanism by which CRHF2 inhibits the expression of mitochondria-mediated apoptosis-related proteins, Western blot analysis was conducted. The results revealed that PM stimulation caused an immediate increase in the expression levels of Bax, Caspase-3, p53, and Caspase-9, and cleaved P poly (ADP-ribose) polymerase (PARP)-like apoptotic proteins (Figure 5). Correspondingly, the levels of antiapoptotic protein Bcl-xL were downregulated. However, concentration-dependent treatment with CRHF2 downregulated apoptotic protein expressions and upregulated the antiapoptotic protein Bcl-xL, thus exhibiting its protective effects.

### 3.7. Protective Effect of CRHF2 on PM-Induced Zebrafish Embryo Survival and Heartbeat Rate

**Figure 6:**
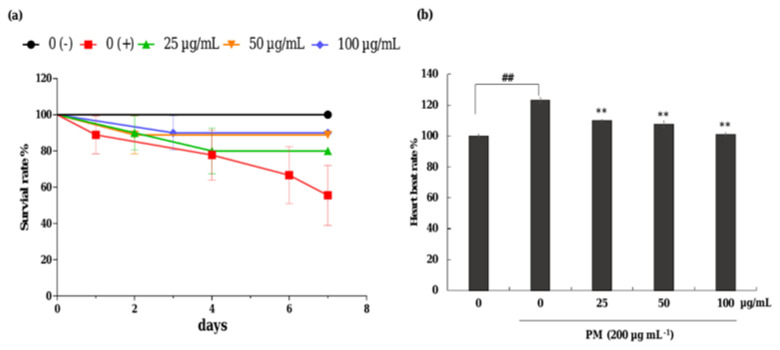
Effects of CRHF2 on PM-induced alterations in survival rate and heart beat rate of zebrafish. (**a**) survival rate, (**b**) heartbeat rate. Experiments were triplicated (n = 3). Results are represented as mean ± SD; ** *p* < 0.01. (# denotes significance compared to control while * represents significance compared to PM treated group 0 (+)).

The protective effect of CRHF2 on PM-induced toxicity in zebrafish was evaluated with 25, 50, and 100 µg/mL concentration ranges of CRHF2 due to there being no toxic effect on the embryos. As shown in Figure 6a, the survival rate of embryos was decreased with the treatment of PM compared to the nontreated group. Preincubation with CRHF2 remarkably and dose-dependently increased the survival rate of embryos. Additionally, the heartbeat rate of zebrafish induced with PM was considerably increased compared to the nontreated group (Figure 6b). It was remarkably recovered to normal levels with the treatment of CRHF2.

### 3.8. Potential of CRHF2 on Inhibiting PM-Induced ROS Accumulation, Lipid Peroxidation and Cell Death in Zebrafish

**Figure 7:**
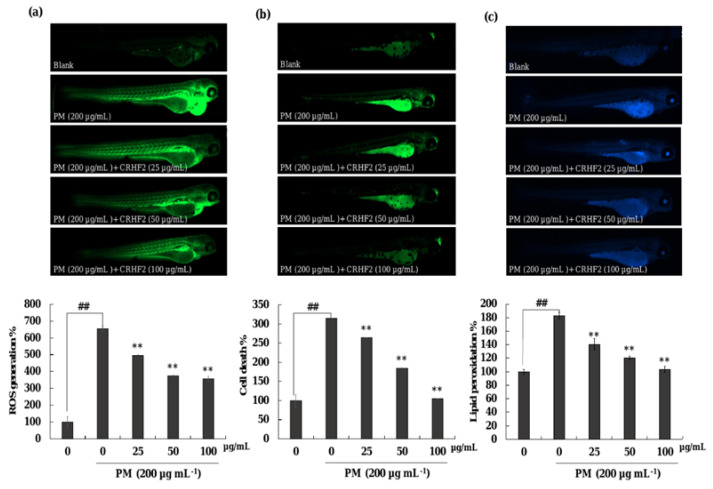
Protective effect of CRHF2 on PM-induced damage in zebrafish. (**a**) ROS production, (**b**) cell death, and (**c**) lipid peroxidation. Experiments were triplicated (n = 3). Results are represented as mean ± SD; ** *p* < 0.01. (# denotes significance compared to control while * represents significance compared to PM-treated group).

As shown in Figure 7a, the ROS production in PM-induced zebrafish was increased compared to that in nontreated fish. However, CRHF2 treatment at 25, 50, and 100 µg/mL decreased the intracellular ROS levels. Additionally, PM stimulation resulted in significant cell death and lipid peroxidation in zebrafish (Figure 7b,c). Similarly, the results showed a decline in lipid peroxidation and cell death in zebrafish following dose-dependent CRHF2 treatment.

## 4. Discussion

With increasing environmental pollution, airborne PM has been identified as the main source of air pollution worldwide. Industrial expansion, coal-burning power plants, vehicle traffic, sandstorms, and other natural events contribute to high levels of PM in the environment [27]. Fine-dust PM has been identified as a major cause of skin diseases, including oxidative stress, inflammation, androgenic alopecia, extrinsic aging, and skin cancers [28]. PM damages the skin by penetrating keratinocytes and causing oxidative stress, which leads to cell apoptosis [28]. Therefore, it is necessary to identify potential treatment strategies for counteracting PM-induced diseases in humans. Recent trends in medicine and cosmeceuticals have identified natural compounds extracted from seaweeds, as safe treatment methods owing to their natural origin. The increasing requirement for potential new substances for the treatment of diseases has highlighted the applicability of these bioactive compounds in the pharmacology, medicine, and cosmeceuticals sectors [29]. Hence, this study was conducted to test the antioxidant and antiapoptotic activities of clionasterol-rich fraction of *C. racemosa* extract against PM-induced oxidative stress, thereby reducing skin damage. *C. racemosa* is a known source of several bioactive constituents such as squalene, having antioxidant and anti-inflammatory activities [30]. The importance of sesqiterpene caulerpenyne isolated from *C. racemosa* was reported earlier [31]. In the present study, fractionation of CRE using hexane resulted in five fractions. Among these, CRHF2 was proven to have superior activity. GC/MS analysis identified clionasterol as the key enriched compound in CRHF2. Several studies have reported the bioactivity of clionasterol [32,33]. In this study, the HaCaT cell culture system was used as an in vitro model and the zebrafish model was used as an in vivo model.

Reactive oxygen species (ROS) represent the major agents of oxidative stress which can be beneficial or deleterious to cells. ROS are small, short-lived, and highly reactive molecules that are important in the regulation of normal physiological functions at low doses. Excess ROS generation in living cells has detrimental effects on proteins, DNA, and membranes [34,35,36]. ROS can be involved in the initiation of oxidative processes as well as in the development of skin diseases. Even though harmful effects of ROS are attenuated by endogenous antioxidants, increased or prolonged presence of ROS interferes with the ROS defense mechanisms in the body. This leads to the activation of cellular responses, which results in the development of numerous skin disorders [33].

The present study findings showed that PM stimulation decreased the HaCaT cell viability, whereas CRHF2 reversed the effect of PM in limiting cell viability. Moreover, intracellular ROS levels increased with PM stimulation; however, they were considerably downregulated with dose-dependent CRHF2 treatment, proving excellent ROS scavenging activity. These findings are similar to those of several previous studies conducted to determine the effect of PM-induced oxidative stress, confirming that PM induces ROS generation in living cells, whereas it is reversed by the treatment with seaweed extracts [37,38].

Elevated ROS levels rework the cellular redox potential and trigger apoptosis [39]. In the present study, PM-induced apoptosis in HaCaT cells was detected using Hoechst 33342 staining, ethidium bromide and acridine double staining, and cell-cycle analysis using flow cytometry. In line with previous studies, Hoechst staining revealed that HaCaT cells exhibit a clear apoptotic morphology of fragmentation and condensation of the nucleus in PM-stimulated cells, which decreased with dose-dependent CRHF2 treatment. Apoptotic nuclei were identified based on the binding of Hoechst to DNA. Therefore, viable cells are indicated as homogeneously stained, round, intact nuclei, whereas apoptotic cells are indicated as fragmented and chromatin-condensed cells. In addition, nuclear double staining allows for the characterization of viable cells and cells undergoing early and late apoptosis. A homogenous nucleus is stained green, and a fragmented nucleus is stained green and orange [40]. In our study, double staining indicated the presence of necrotic cells in PM-stimulated HaCaT cells and indicated that a high number of cells had a clear apoptotic morphology with fragmented nuclei. Pretreatment with CRHF2 ameliorated PM-induced apoptosis by decreasing the number of apoptotic cells. This treatment also restored chromatin condensation and fragmentation in a dose-dependent manner. This phenomenon has also been demonstrated in an earlier study on antiapoptotic effect of Eckol isolated from *Ecklonia cava* against PM-induced cells [41]. To investigate the cell-cycle phase, flow cytometry was performed using propidium iodide staining. Similar to results obtained in an earlier study, prominent sub-G1 accumulation was observed in PM-stimulated HaCaT cells [42]. However, a reduction in sub-G1 phase cell population after CRHF2 treatment clarifies its antioxidant potential.

PM triggers apoptosis by the following two main pathways; the mitochondria-mediated intrinsic pathway and cell surface receptor-mediated extrinsic pathway. The mitochondria-mediated apoptosis pathway is a major signaling pathway resulting in apoptosis due to its sensitivity to a high number of death stimuli. Increased ROS generation in mitochondria results in mitochondrial damage as well as the activation of mitochondria-mediated apoptosis. Therefore, the effects of PM on mitochondrial ROS generation were also tested in this study, using the DHR123 staining method. Excessive mitochondrial ROS results in the activation of mitochondrial-mediated apoptosis pathway [43]. Mitochondria-derived ROS targets nearby structures such as mitochondrial DNA (mtDNA), further increasing ROS generation, leading to loss of mitochondrial membrane potential. This results in apoptosis through the mitochondrial pathway. In our study, compared to nontreated cells, PM stimulation increased ROS generation in mitochondria; however, CRHF2 regulated the mitochondria in a stable state.

The mitochondria-mediated apoptosis pathway is controlled by a complex network of signaling cascades. Therefore, Western blot analysis was carried out to assess the effect of CRHF2 on mitochondria-mediated apoptosis pathway protein expression. PM stimulation initiates this pathway by the permeabilization of the outer mitochondrial membrane. This is controlled by Bcl-2 family proteins, which include antiapoptotic proteins (Bcl-2 and Bcl-xL) and proapoptotic proteins such as Bax [44]. The activation of Bax and inhibition of Bcl-xL promote the opening of mitochondrial permeability transition holes, thus causing the release of cytochrome C into the cytoplasm. This activates caspase-9 and caspase-3 protein expression, resulting in apoptosis [45]. The permeabilization of the mitochondrial membrane by PM also results in the activation of p53, leading to apoptosis [46]. PARP inhibits its catalytic activity, which ultimately provokes cell apoptosis. In our study, PM treatment resulted in the alteration of Bax and Bcl-xL protein levels in cells, and it ultimately increased the expression levels of caspase-3, caspase-9, p53, and cleaved PARP. Similar results were observed in a study on the effect of PM on endothelial cells [41]. Our study findings suggest that PM initiates mitochondria-mediated apoptosis protein expression, leading to cell damage. Results showed that dose-dependent treatment of CRHF2 inhibits the expression of proapoptotic proteins, attenuating the negative effects of PM in skin keratinocytes. Similar results were obtained in a study on the antiapoptotic activity of dioxinodehydrockol, a phlorotannin isolated from *E. cava* [47].

The zebrafish model is becoming popular as an animal model for testing human disease conditions. Therefore, in this study, it was used as an in vivo model to investigate the antioxidant activity of CRHF2. ROS production levels measured in zebrafish indicated that CRHF2 treatment resulted in declining intensities, revealing its protective effect against PM-induced ROS generation. Moreover, DPPP staining indicated the intensity of lipid peroxidation due to PM stimulation in zebrafish. ROS stimulated the lipid peroxidation, resulting in the disruption of membrane lipid bilayer and membrane-bound receptor activities. This leads to an increase in tissue permeability [24]. Lipid peroxidation results in protein inactivation by increasing unsaturated aldehydes through crosslinking [9]. Moreover, results indicated that cell death in zebrafish was caused by the accumulation of important macromolecules, such as lipid peroxidases, due to ROS generation. However, CRHF2 protected zebrafish against PM-induced oxidative stress through ROS scavenging. The results obtained in this study are well consistent with those of previous studies on polysaccharide extracts from *Hizikia fusiforme* and *Padina boryana* [25,48]. Our results indicate that *C. racemosa* extract possesses strong antioxidant activity, as demonstrated by the decline in cell death, ROS production, and lipid peroxidation in the zebrafish model.

## 5. Conclusions

In conclusion, the present study demonstrated that PM induced oxidative stress-mediated cell apoptosis, and the hexane fraction isolated from *C. racemosa*, CRHF2, attenuated PM-induced skin damage by inhibiting ROS generation and activating the mitochondrial-mediated apoptosis pathway in HaCaT cells. The apoptosis was inhibited by regulating apoptosis pathway proteins and DNA damage in PM-induced cells. Furthermore, CRHF2 suppressed ROS production, cell death, and lipid peroxidation in PM-treated zebrafish, suggesting its potential use in attenuating oxidative stress in vivo. These results suggest that *C. racemosa* possesses strong antioxidant and antiapoptotic activities and could be a promising ingredient in the pharmaceutical and cosmeceutical industries.

## Data Availability

Data are contained within the article.
